# Social Structure in a Roma Settlement: Comparison over Time

**DOI:** 10.3390/ijerph17197311

**Published:** 2020-10-07

**Authors:** Michal Kozubik, Daniela Filakovska Bobakova, Rastislav Rosinsky, Martina Mojtova, Miroslav Tvrdon, Jitse P. van Dijk

**Affiliations:** 1 Department of Social Work and Social Sciences, Faculty of Social Sciences and Health Care, Constantine the Philosopher University in Nitra, 949 74 Nitra, Slovakia; mkozubik@ukf.sk (M.K.); mmojtova@ukf.sk (M.M.); mtvrdon@ukf.sk (M.T.); 2 Department Community & Occupational Medicine, University Medical Centre Groningen, University of Groningen, 9713 AV Groningen, The Netherlands; 3 Olomouc University Social Health Institute, Theological Faculty, Palacky University Olomouc, 771 11 Olomouc, Czech Republic; daniela.filakovska@upjs.sk; 4 Graduate School Kosice Institute for Society and Health, Faculty of Medicine, Safarik University, 040 01 Kosice, Slovakia; 5 Department of Health Psychology and Research Methodology, Faculty of Medicine, Safarik University, 040 01 Kosice, Slovakia; 6 Institute of Roma Studies, Faculty of Social Sciences and Health Care, Constantine the Philosopher University in Nitra, 949 74 Nitra, Slovakia; rrosinsky@ukf.sk

**Keywords:** Roma, social structure, comparison, 18th century, 21st century, Slovakia

## Abstract

The objective of the present study was to compare the social structure and internal establishment of a Roma community in two historical periods: in the 18th century and the present. We analysed Samuel Augustini ab Hortis’s work, “*Von dem Heutigen Zustände, Sonderbaren Sitten und Lebensart, Wie Auch von Denen Übrigen Eigenschaften und Umständen der Zigeuner in Ungarn*” (On the Contemporary Situation, Distinctive Manners and Way of Life, as Well as the Other Characteristics and Circumstances of Gypsies in Greater Hungary), written in 1775–1776. Using content analysis, we subsequently compared his findings with our recent data from analogous qualitative research in a geographically-defined area of north-eastern Slovakia, the same region in which Augustini lived. Data collection was intensely conducted in 2012–2013 and once more in 2017–2019. The qualitative methods included direct observation, semi-structured interviews and focus groups. Four key informants and more than 70 participants collaborated in the study. The greatest difference we observed compared to the 18th century was the absence of a leader of the community, a “vajda”, whose status was taken over by a new social class of “entrepreneurs”. The most vulnerable group of the segregated and separated Roma communities are the “degesa”, the lowest social class. They face a phenomenon consisting of so-called triple marginalization: they live in one of the most underdeveloped regions of the country, they inhabit segregated settlements and they are excluded by their own ethnic group. The socioeconomic status of the richest classes has changed faces, while the socioeconomic status of the lowest has not. We found a misconception among helping professionals (e.g., social workers) regarding the homogeneity of the Roma community. This calls for more attention to the erroneous use of the ethnic-based approach in the helping professions.

## 1. Introduction

The Roma population is the largest transnational ethnic minority in Europe, characterized by linguistic [1,2,3], cultural [4,5] and historical heterogeneity [6,7,8]. Roma are thus not a single, homogeneous group of people [9,10,11,12,13]. Many aspects of Roma culture have changed over the centuries, but some of them are still the same [14]. We know of comparisons of various areas of public health with regard to Roma, such as housing, nutrition, death and illnesses [15,16,17,18], in different historical periods. However, these studies lack information on the internal social structure of geographically-defined Roma settlements in such historical comparison. For this reason, we decided to focus on the internal establishment and social structure of settlements (towns, village peripheries and segregated settlements) and to compare them with their descriptions from approximately 250 years ago. Furthermore, we also attempt to outline a stratification model of the inhabitants of one particular settlement.

**Figure 1:**
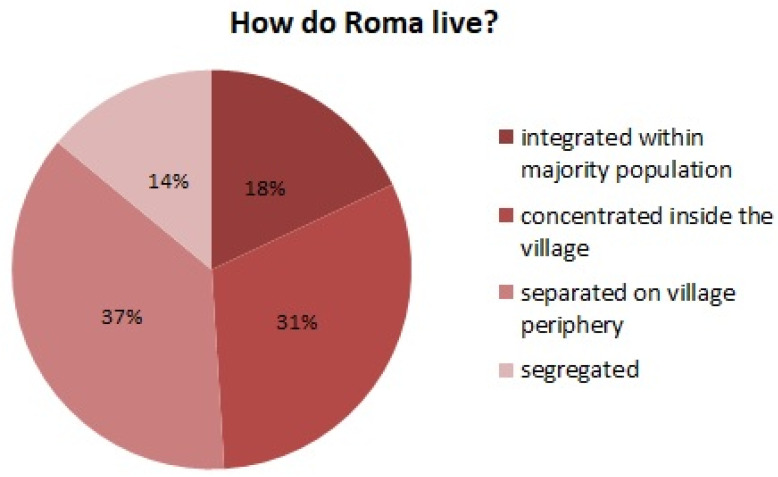
How do Roma live? (Slovakia). Source: Kerekes, 2019 [20].

Currently, according to qualified estimates, there are about 440 thousand Roma in the Slovak Republic [19], which is about 8% of the total Slovak population. They live either integrated within the majority population or scattered in groups with various degrees of concentration (Figure 1).

Of the estimated 440,000 Roma living in the Slovak Republic, 332,000 are settled in 1052 concentrations separately from the majority population. The spatial and social distance between Roma and the majority population has existed for centuries. Government policies and legislation have contributed significantly to the situation. In the 1950s, Law No 74/1958 on Permanent Settling of Nomadic Persons banned the nomadic way of life [21]. The transition from a totalitarian state towards a democratic society which started in the 1990s plunged the Roma community into even greater social isolation. The changes post-1989 caught Roma unawares, and the Roma population was not prepared for the new situation. The current position of the Roma in Slovakia is also influenced by developments in the Czech Republic since the separation in 1993, the pre-1989 history of Czechoslovakia and the transition to democracy and market capitalism thereafter. The Communist regime’s policies before 1989 regarding the living conditions, education and working patterns of the Roma continued, determining the growth potential of the Roma communities. The changes which took place after 1989 have resulted in social stratification of the Roma population, and this has crucially affected their way of life [22,23].

The social structure of the various types of Roma communities has been described in terms of social exclusion [23,24]. The European Commission [25] defined exclusion in terms of limited access to the resources necessary for participation in social, economic and political life. In the context of social exclusion, a number of dimensions are identified [26,27,28]: an economic dimension (long-term unemployment, income poverty, dependence on social benefits and participation in alternative ways of subsistence), a social and cultural dimension (relation to the majority population, discrimination in school environment, health care and unemployment), and a political dimension (lack of political representatives, low participation in elections and low participation rate in dealing with own situation). Furthermore, a community dimension is identified (collapse of support networks, unavailability of social services and devastation of the surrounding environment), an individual dimension (deteriorated health status, high morbidity, low level of acquired education and qualification and loss of confidence and self-esteem), and a spatial dimension (concentration of excluded groups and communities in specific locations which can be considered as disadvantaged in terms of location, lack of infrastructure and unavailability of services) [29]. In the case of the marginalized Roma communities in Slovakia, we can talk about the phenomenon of double marginalization. This means that Roma not only live in segregated settlements, but also in the regions with the highest unemployment rate [24]. 

Another way to perceive social exclusion is the concept of the culture of poverty [30]. This concept states that identical patterns of behaviour are manifested in the value system and attitudes, family structures, relationships and customs of a poor community, regardless of ethnicity, nationality or geographical space. Manifestations of the culture of poverty can appear as illogical to uninformed observers, but they are highly useful for understanding the daily life of the inhabitants of poor settlements. The culture of poverty has reproductive potential and is passed on from one generation to the next [31]. Social distances can be defined subjectively (attitudes towards various groups of persons) and objectively (meetings within friendship and partnership networks). Usually, the definition is within the economic field [32]. The distance defines the social interaction and relationship with other people and characterizes personal and social relationships [33].

Comparative studies which historically describe the internal establishments in settlements are completely lacking. The objective of the present study is to compare the internal establishment of the Roma communities described in the past [34,35] with the present situation. The intention is to draw attention to the changing internal establishment and societal structure within the Roma communities, as well as to the related risk of ethnic-based approaches in the helping professions [36].

## 2. Methods

The initial phase of this study consisted of detailed analysis of the texts issued in 1775–1776 by Samuel Augustini ab Hortis (1729–1792), entitled “*Von dem Heutigen Zustände, Sonderbaren Sitten und Lebensart, wie Auch von Denen Übrigen Eigenschaften und Umständen der Zigeuner in Ungarn*” (On the Contemporary Situation, Distinctive Manners and Way of Life, as Well as the Other Characteristics and Circumstances of Gypsies in Greater Hungary). Augustini published this work as a series of articles in the magazine *Kaiserliche Königliche Allergnädigste Privilegierte Anzeigen aus Sämstlichen Kaiserl. Königl. Erbländer* during the reign of Empress Maria Theresa (1717–1780) in the Austro-Hungarian Empire (Slovakia was part of the Empire at that time). He studied Lutheran theology in Wittenberg (Germany) and mathematics and algebra in Greifswald (Germany). After his studies, he came back home and worked as a teacher and later as rector of the Lutheran lyceum in Kezmarok (Slovakia). He took interest not only in Roma culture, but also in botany and mineralogy. To ensure the highest possible validity of our results, we decided to use compatible qualitative research methods exactly as they were used by Samuel Augustini. 

### 2.1. Samples

**Figure 2:**
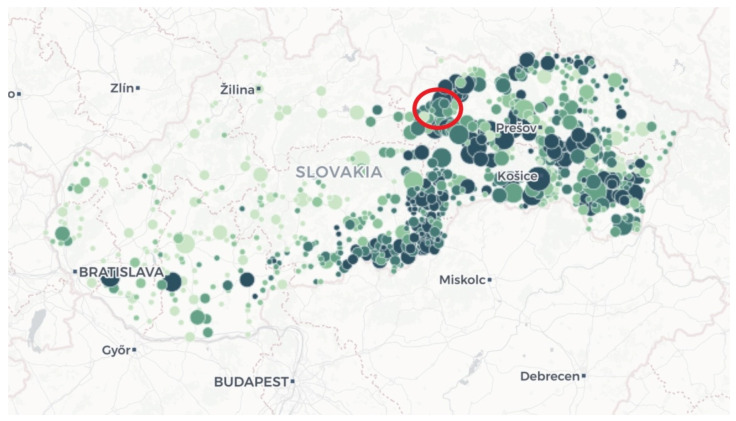
Where do Roma live? Slovakia, showing the district of Poprad (red circle). Source: Kerekes, 2019 [20] (green dots: the bigger the circle, the larger the number of Roma inhabitants, and darker colour indicates increased Roma population density) and the authors.

**Figure 3:**
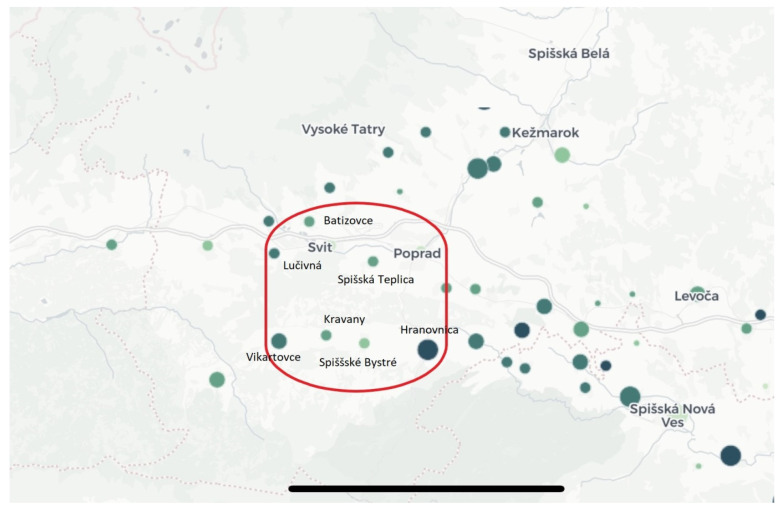
Data collection sites (Poprad, Spisska Teplica, Hranovnica, Spisske Bystre, Vikartovce, Kravany, Lucivna, Batizovce) adapted from Kerekes, 2019 [20]. For the larger and darker circles see Figure 2.

The data was collected in the same area in which Samuel Augustini ab Hortis lived almost 250 years ago: the district town of Poprad, located in the north-east part of Slovakia, in the region known as Spis. Compared with the total number of Roma throughout Slovakia (8%), a greater proportion of Roma (30%) live in this region [19]. We included all types of respondents: integrated with the majority population (the town of Poprad and the town part of Spisská Teplica), concentrated within the village (Spisske Bystre), separated in the village periphery (Kravany, Vikartovce, Lucivna, Mengusovce) and segregated (Spisske Bystre, Hranovnica, Spissky Stiavnik). The number of inhabitants in these concentrations varies from 270 in Kravany to 1734 in Hranovnica [19] (Figure 2 and Figure 3). The Roma in our sample are Rumungre (a Roma subethnic group living in the eastern part of Slovakia), which we point out as conditions might differ between this group and Vlachico Roma (residing mostly in the southern and western parts of the Slovak Republic). We worked with four key informants, interviewed more than 70 Roma people, and organised two focus groups. Each group consisted of 20 informants. The participants in both groups were chosen randomly and met certain criteria: they had to identify themselves as Roma and had to live in a segregated settlement. All of them lived in extreme poverty and were dependent on welfare benefits. We recorded more than 1700 min of interviews and took many photographs. All the addressed people confirmed their ethnicity and voluntarily consented to participate in the study through audio recordings [14,15].

### 2.2. Data Collection

The initial phase of the study included content analysis of the historical documents forming Augustini’s work. We visited Poprad and Spisska Sobota. Samuel Augustini ab Hortis himself lived and worked in the same locality. In the analysis of his work, we focused on the sections describing the internal establishment of the life of the Roma in the 18th century. Based on the files, before the field research and interviews, we prepared a detailed history. Before going out into the field, we contacted four key informants who came directly from the areas surveyed. The first intensive field research took place in the summer months of 2012–2013. During these and later on in other, later phases of data collection (2017–2019), we ourselves lived in a separate locality at the end of the village. We visited every type of settlement and in each we randomly selected several informants. Afterwards, we gradually included all the interested persons we acquired through the snowball technique. The information the Roma provided to us were recorded in a field logbook and on a voice recorder. We present the most important quotations as word-for-word translations of the recordings.

### 2.3. Analyses and Reporting

Contemporary political, cultural and scientific stimuli significantly influenced Augustini in writing his major research monograph. It was mainly an attempt by Empress Maria Theresa to find a solution to the so-called Roma issue in the Kingdom of Hungary, which must be perceived as an organic part of Enlightenment-absolutist reforms. Their basis was the theory about the number of citizens as an indicator of the country’s wealth. According to this theory, it was necessary to make the Roma (in German *Zigeuner*) useful citizens in the Kingdom of Hungary. Augustini based his work on several resources. The foundation consisted of contemporary and older literature and manuscripts. He worked with dozens of resources, referred to them in the text and recorded them accurately in the reference list. Augustini regarded the views of individual authors critically and rejected their romantic ideas, which was revolutionary for the given period. He rejected non-professional names and was inclined to those he considered to be correct, based on his observations and reflections.

Our efforts were directed at using content analysis to process Augustini’s written work in detail. We created the principal categories by conceptualizing the data and applying open coding. In our analysis, we were inspired by the work of Weber, who also influenced the school of the new ethnography by Geertz [37,38,39], which was our paradigm and model. We compared Augustini’s central categories with the current perceptions of social structure and internal establishment from the Roma perspective. For the purposes of this study, we focused exclusively on these two areas.

### 2.4. Research Ethics

In our research, neither animals nor plants were studied. Human beings from 1775 to 1776 were not studied by us, and human beings in 2012–2013 and 2017–2019 were studied in line with the Helsinki Declaration. All the participants agreed to their participation in the study. Their informed consent was obtained and archived through audio recordings.

## 3. Results

We focused on the social structure and internal establishment of the Roma community described in 1775–1776 by Samuel Augustini ab Hortis. We compared his writings with the current situation in the various types of Roma settlements. We added our informants’ verbatim statements. They were translated into English by the authors. The results for the historical periods are presented separately to ensure better transparency.

### 3.1. Social Structure of a Roma Community before and in Augustini’s Period

Augustini [34,35] considered the social establishment of all Gypsies in what was then the territory of Hungary to be the same. He attributed the leadership position in the community to the vajda. He describes the vajda’s position vis-a-vis the authorities and his relationship with his own people. In addition to the term vajda, the expressions duke, prince or king were used to refer to a leader. Augustini states that Gypsies came to Germany with Duke Michal, and to Italy with Duke Andrej, and they were brought to Bavaria by King Zindanel. He claims that their titles served for external effect and communication with the authorities.

**Figure 4:**
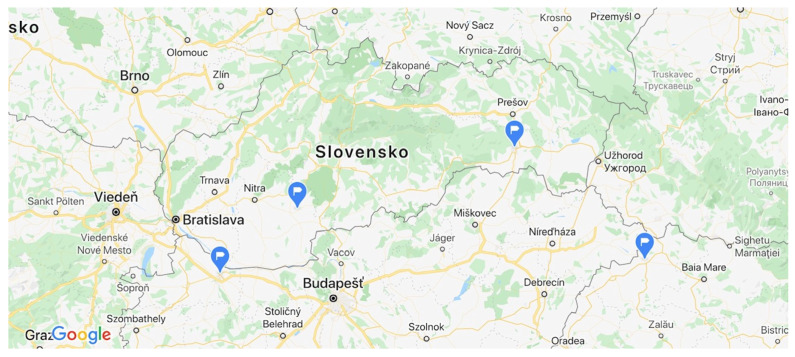
Locations of four Roma *vajdas* (dukes): Györ, Košice, Levice, Satu Mare (blue pointers). Source: Google Maps, 2020 and the authors.

In Hungary four dukes were present, on both sides of the Danube and the Tisa. They were located near Györ (Hungary), in Levice and Košice (Slovakia) and near Satu Mare (Romania) (Figure 4).

**Figure 5:**
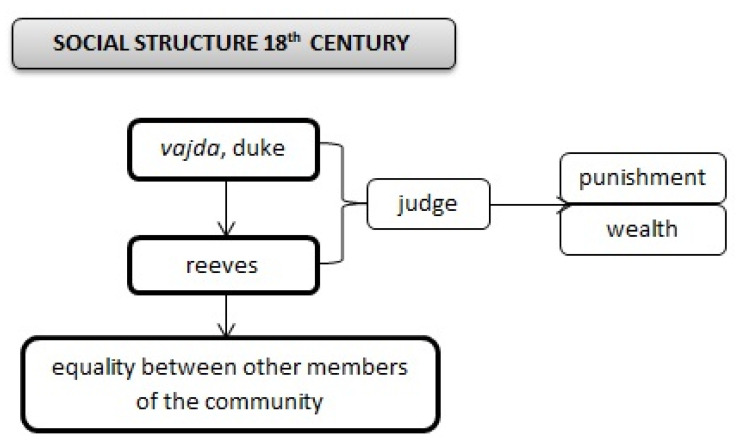
Roma social structure in the 18th century.

This power structure was approved by Imperial authority. When there were more Gypsies in an area, they elected a reeve from their own people. However, he did not have as much power as a “vajda”. In the election of a reeve, his abilities, intelligence and skills were not considered. His wealth and clothing were more important. He was expected to be of middle age and to outdo the others in his appearance. He was characterised by a proud gait, a big whip and his facial expression was serious and wise (Figure 5).

The duty of the Gypsy reeve was to supervise his subordinates, and prevent the disappearance of annual benefits and taxes during their collection for the prince’s chamber. In addition, he was permitted to present the actions and complaints of his people in an appropriate place. When his subordinates were suspected of theft, all the tents could be searched on his request to see if stolen items were there. If they were found, they had to be returned. The culprits were whipped by the Gypsy reeve. The reason for the physical punishment was the satisfaction of the plaintiff and the; warning to their own people. The rest of the community, except for the leaders (synonymous naming vajda, duke, reeve), was equally well served by strong social cohesion. Roma were perceived by Augustini as a homogeneous group of people. 

### 3.2. Social Structure of Roma Community at Present

The social structure nowadays has changed considerably from what is described above. In the settlements there are three classes of population: “entrepreneurs”, “middle-class” and “degesa”. The most powerful class is made up of the so-called “entrepreneurs”, who prosper at the expense of the poorest residents of the settlement. Their activities include usury, job offers and other activities. The entrepreneurs are not perceived negatively by the residents of the settlement. On the contrary, the poor often perceive them as persons who help them in case of emergency, if social support by the family fails. Usury adapts to the deteriorating material conditions of the settlement as a whole (Box 1).

Box 1 **About entrepreneur’s behavior.**

“There is usury but not in the form of money. It is food aid. The usurers try to sell food as expensively as possible and then reclaim it from the borrowers in the form of cash benefits that poor Roma receive from the government. It is a regular activity, it is a vicious circle, and the poor cannot get out of it. The usury also happens in a way that they buy a litre of wine for 1.30 EUR and the borrower must pay 3.40 EUR. Nowadays it has all re-focused on food; it’s a better business for them (usurers). Tobacco, cigarettes and wine are important” (male; low SES; age 30 years).

The entrepreneurs have contacts with various construction companies, including the foreign ones, and provide transport for a commission. This is usually a case of undeclared work, which is often inadequately paid [40].

The “middle class” consists of people working in different sectors: low-skilled labour, seasonal workers and moonlighting jobs within the grey labour market. Some of them are social workers, teachers’ assistants and community workers for the Roma minority. The middle class keeps both the entrepreneurs and degesa at a distance. The Roma middle-class shows some understanding of the contempt felt by the non-Roma majority for poor Roma (degesa), and at the same time some disapproval of the illegal practices of the entrepreneurs (Box 2).

Box 2 **About other Roma.**

“Some of our Roma would tell me, you are not a Roma (anymore), you’ve become a non-Roma. I can excuse poverty, yes, but I do not like the dirt… I would imagine it like this: It doesn’t matter that I am a Gypsy woman. I wish that these poor Roma were not here. ‘Bang’ and they would be gone. These people are never going to become better. When they cook, they take a bucket with waste and do not put it in the garbage container; they make a mess. I suffer for those mistakes they make” (female; middle-class; age 46 years).

Degesa, paradoxically, maintain the greatest distance from other degesa. There are cases of fraud and theft among the poorest people, even within broad families, which gainsays the Roma proverb: “Roma are not robbed by Roma.” The most frequently stolen commodity is wood, the most valuable raw material in the winter season. The winters are very cold in the studied territory, and wood is the only fuel Roma can imagine (Box 3).

Box 3 **Poor Roma about other poor Roma.**

“They steal…when their parents send them as they have nothing to burn. It happened to me that a small kid, 10-12 years old, stole a sheet of metal which I had out the back” (male; low SES; age 35 years).

“Roma people help each other, but on the other hand, they also beat each other. I’ll help you with wood, but in the morning, you’re not going to have it there…” (male; low SES; age 32 years).

**Figure 6:**
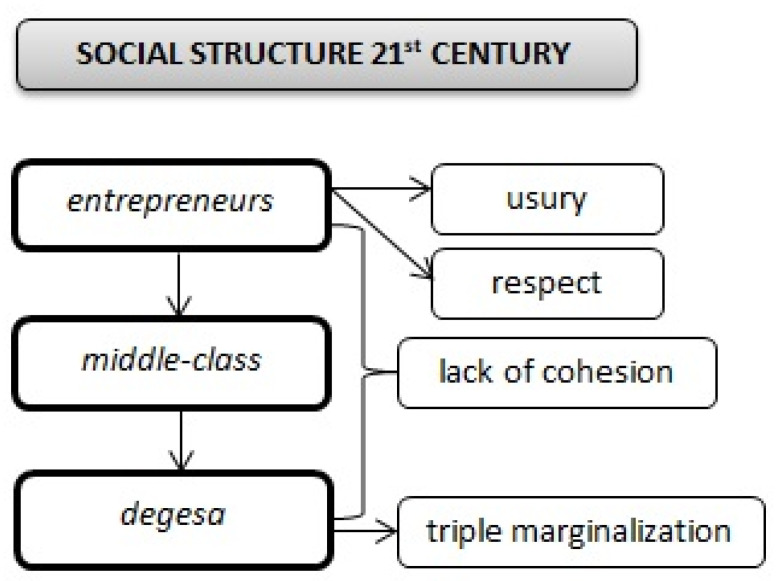
Social structure nowadays.

The distance between the various classes of the Roma community was evident whenever the researchers entered the settlement. This environment is characterized by a high degree of social stratification. The rich do not meet with the poor, though business issues are an exception. All these classes, however, are seen by the majority population as homogeneous, and perceived negatively [41]. These relations are influenced by the size and the number of people in the settlement (Figure 6). 

## 4. Discussion

We compared the social structure and internal establishment of the Roma community in two periods: the 18th century and the present. We found that the structure of the Roma community has changed considerably. 

In the past, the leadership in the Roma community was represented by the “vajda”, also known as duke, prince or king. According to the Imperial decree of 13 November 1761, the position of “vajda” was abolished. A new leader position was created: the reeve. He was elected from and by the Roma themselves, and his election was approved by a local institutional, political authority. The reeves were to visit the Roma every Thursday and check whether they were adhering to the decrees [42]. All decrees issued by Maria Theresa and Joseph II regarding the Roma were published between 1760 and 1784. They were sent from the Hungarian Royal Governing Council to individual districts, which were the executors of the decrees. Until the middle of the 18th century, the Roma belonged under the authority of the main regional governor, but from that point on the local landlords became their masters [43]. After centuries, the positions of leader, the “vajda” as well as the reeve, completely vanished from the area of north-eastern Slovakia, where Samuel Augustini lived and worked. They were taken over by an elite group called “entrepreneurs”; they often prospered from the unfavourable social situation of the lowest class of “degesa” through the use of various modes of usury (cash and food) [44]. On the other hand, the position of leader, vajda or king, is still present in the Vlachico Roma communities prevalent in western and southern parts of the Slovak Republic [45]. While even in the recent past, relations within extended families or larger family-ancestral formations (“fajtas”) in segregated Roma settlements in eastern Slovakia were characterized by generalized reciprocity and mutual solidarity [46,47], today, the applicability of these models needs to be reassessed, especially in cases involving successful usurers in segregated rural settlements [44].

In the 18th century, members of the Roma community were equal, apart from the leaders described above. At present, the social functioning does not differ between the individual types of settlements (segregated settlements, concentrations within and at the periphery of villages). The exception are the integrated Roma families living scattered among the majority population of the district town of Poprad. Integrated Roma are usually part of the middle-class in the general population. However, there is also a “middle-class” in the settlements. These are not as poor as degesa, but on the other hand not as well off as the majority middle-class. This class is characterized by a relatively high rate of employment among its members. This class in urban as well as rural areas consists of people working in various sectors: low-skilled labour, seasonal workers or moonlighting jobs within the grey labour market. Existing measures regarding the high unemployment rate of Roma do not so far reflect the heterogeneity of this community in detail [48]. 

The most difficult living conditions are reserved for the lowest class: “degesa”. In the traditional Roma caste system, the degesa do not adhere to the rules of ritual purity and other standards. Some degesa families, for example, allegedly consume unclean types of meat [49]. We agree that poverty and social exclusion lead to multiple marginalization. In any case, it is not just double marginalization [23,24]. Based on our data, we suggest extending this concept to include the phenomenon of so-called triple marginalization, by which we refer to the communities living in the part of the country with the highest unemployment rate, living segregated from the majority and composed of part of the population which is excluded within their own ethnic group. The majority of functional social networks in socially-excluded Roma communities have the character of closed primary networks with prevailing strong ties. Such social networks are an ideal space for acquiring an important feeling of personal security. In this space, however, individuals have minimum prospects of obtaining new resources, which significantly restricts their social mobility and flexibility from the point of view of their achieving social inclusion, once identified as socially excluded [50].

### 4.1. Strengths and Limitations

The uniqueness of the present study lies in the hitherto unconducted comparison of the social structure of the Roma settlements in very different time periods (the 18th century and the present). The benefit is the perspective provided exclusively by the inhabitants of separated and segregated settlements. The study answers the question: how has the internal establishment and social structure of the Roma community changed compared to the past? In 2012–2013 and 2017–2019, multiple ethnographic materials were collected, enabling a coherent comparison of Augustini’s work with the present situation of Roma communities in the eastern parts of the Slovak Republic. 

The limitations of our findings lie the inability to generalize the results for the whole Roma population, and the data reliability. Verification can be problematic because the environment of the settlements is a living organism. Despite the idiographic approach used and our avoidance of any generalization, based on our experience we may conclude that the conditions and variables affecting the lives of the inhabitants of the settlements remain as if preserved in time; they change only slowly and thus do not affect the data we collected. The results appear typical for the areas of the sub-Tatra region, Spis and eastern Slovakia. Social structures may vary based on the size of communities, and moreover the situation might be different among the *Vlachico* Roma living in western and southern parts of the Slovak Republic. 

### 4.2. Implications

The focus here is multidimensional: from contemporary historical descriptions to the social structure of the communities, which can be used as a source of information for field workers, as well as for policymakers at meso and macro levels. The social structures of Roma settlements are changing, and many myths are circulating in society. Part of these myths has its origins in the past. This also applies to the helping professions. For this reason, we try to point out the changing character of social structures and their heterogeneity between communities and within communities, which should be taken into account by the helping professions when entering communities with good intentions, because misunderstanding and disrespect of social structures can lead to application of bad practices.

In Romological discourse, deconstruction of the stereotyped ethnicity-based perspectives on marginalized Roma communities is recommended [36]. By remaining unchanged, policy documents, technical articles and teaching texts and books merely strengthen prejudices and educate future helping professionals, including social workers, inappropriately. In addition, the conceptualization of Roma in these documents may lead to an incorrect understanding of their being a homogeneous group of people. The supposed homogeneity of the Roma is furthermore associated with a number of negative phenomena such as poverty, segregation, or dependence on social benefits (reference—Podolinska, Skobla), which, together with unfamiliarity with structural determinants, as well as historical and political contexts, reinforces unfavourable societal discourse. [36,51]. 

## 5. Conclusions

In summary, our comparison of the social structure and internal establishment of Roma communities in two historical periods has revealed considerable differences. They include mainly the change in the internal community establishment. While in the second half of the eighteenth century, there was a “vajda” at the head of a group, and later each larger Roma community elected a reeve, who was a sort of local authority potentially linking the majority and Roma with some actual power on a local level, in the present day, the privileged positions are held by rich family clans respected by the rest of the community, who are often engaged in usury and possess economic and social power. The socially lowest class is the group of “degesa”, who are seriously threatened by triple marginalization; on top of double marginalization (geographic and economic), these people also face exclusion within their own ethnic group. This form of social structure is typical, although not universal for the segregated settlements and separated groups. In addition to these findings, we draw attention to the still-existing stereotyped ideas of the homogeneity of Roma society. Furthermore, we question the usefulness of ethnicity-based approaches for the helping professions.
